# *Dendrobium* Multi-Omics Reveal Lipid Remodeling in Response to Freezing

**DOI:** 10.3390/metabo12121216

**Published:** 2022-12-03

**Authors:** Xinqiao Zhan, Yichun Qian, Bizeng Mao

**Affiliations:** 1 Institute of Biopharmaceuticals, Taizhou University, Taizhou 318000, China; 2 Institute of Biotechnology, Zhejiang University, Hangzhou 310000, China; 3 Ministry of Agriculture Key Lab of Molecular Biology of Crop Pathogens and Insects, Hangzhou 310000, China; 4 Key Laboratory of Biology of Crop Pathogens and Insects of Zhejiang Province, Hangzhou 310000, China

**Keywords:** *Dendrobium*, lipidome, proteome, cold stress

## Abstract

Freezing damage is a common phenomenon responsible for reduced yields of economic crops. Regulation of lipid metabolism plays an important role in plant growth and adaptation during freezing. We previously carried out transcriptome and untargeted metabolome analyses to determine the regulation of flavonol and anthocyanin biosynthesis during freezing treatment (FT) and post-freezing recovery (FR) in *Dendrobium catenatum*. However, changes in lipid levels are hard to confirm by untargeted metabolomics analysis alone. Regulation of lipid metabolism in response to freezing is largely unknown in *Dendrobium*. In this study, a multi-omics strategy was used to offer a better means of studying metabolic flow during FT and FR. To this end, 6976 proteins were identified by the 4D_label-free proteome, including 5343 quantified proteins. For each of the two conditions, we enriched differentially accumulated proteins (DAPs) into 15 gene ontology (GO) terms, including primary metabolism, lipid metabolism, and photosynthesis processes. We also identified 7 lipid categories and 3672 lipid species using lipidome assays. We found significant remodeling occurring in the phospholipid category during FT and FR. We also found that most sphingolipids were significantly upregulated. An integrated multi-omics analysis revealed significant changes in the expression levels of 141 mRNAs and encoding proteins under both FT and FR conditions. During FT, phospholipase A (PLA) and phospholipase D (PLD) were associated with phospholipid editing and galactolipid remodeling. These results provide valuable new insights into how the freezing tolerance of *D*. *catenatum* might be improved by genetic engineering.

## 1. Introduction

Freezing stress can lead to serious reductions in agricultural yields. Some economic crops are naturally adapted to low temperatures but not sub-zero temperatures, including apples, tea, pepper, spinach, and cucumber [1,2,3,4,5]. Membranes are major injury sites in plants during periods of freezing because low temperature decreases their fluidity [6]. Plants can change their membrane lipid composition in response to freezing conditions and release fatty acids through the degradation of phosphatidylcholine (PC) and phosphatidylethanolamine (PE) [7]. In extraplastidic membranes, freezing increases phosphatidic acid (PA) and lysophospholipid levels [8]. These increase membrane fluidity and mediate signal transduction [7]. The unsaturation of fatty acids in phospholipids in chloroplast membranes is strongly related to nonphosphorus glycerolipid metabolism under cold stress [9]. The two major glycerolipids in the membranes are monogalactosyldiacylglycerol (MGDG) and digalactosyldiacylglycerol (DGDG) [10,11]. A unique anionic glycerolipid, sulfoquinovosyldiacylglycerol (SQDG), is only found in chloroplast membranes. SQDG biosynthetic enzymes are not only involved in lipid remodeling under phosphate starvation but also flavonoid accumulation under abiotic stresses [12,13]. These membrane lipids play important roles in the replacement of phospholipids under cold stress. Chilling alters the compositions of galactolipids and carotenoids, including decreases in the MGDG:DGDG ratio and increased levels of lutein [5]. Plants can enhance their freezing tolerance during cold acclimation. After 14 days of cold acclimation, most natural accessions of *Arabidopsis* accumulate massive amounts of storage lipids, including long-chain unsaturated triacylglycerides (TG) [14]. In comparison with non-acclimated plants, the species of TG (52:0, 58:9), MGDG (34:2, 34:3, 36:7), and glucosylceramide (GlcCer d18:1/c24:0) all increase under cold stress [14]. These specific lipid species are important to the cold tolerance of plants. Peanut cold-sensitive variety FH18 is more severely damaged during 24 h of cold stress. Lipid species increase in FH18, including C36:5-, C36:6-PA, C36:5-, and C36:6-DGDG [15]. Change in lipid composition under low temperature is major in improving postharvest fruit quality. In peaches, low temperature induces ethylene production to promote fruit softening and lipid desaturation [16]. Ethylene-responsive factors (ERF) are important regulators for defense against cold stress [17]. Jasmonic acids (JAs), as lipid-derived compounds, play a key role in plant stress responses and development [18]. Regulation of ERF family genes is related to JA signaling, which contributes to the chilling response in pepper fruit after harvest [4]. Thus, lipid composition has a profound impact on the adaptive responses of plants to their environment.

*D*. *catenatum* belongs to the Orchidaceae family and is widely cultivated as an economic medicinal herb in southeast China. It is valued for its medicinal components, such as polysaccharides, alkaloids, terpenoids, and flavonoids [19,20,21]. Most *D*. *catenatum* plants can survive in greenhouses during winter, but yields are greatly affected by cold, especially when temperatures fall below zero. After freezing stress, the leaves of *D*. *catenatum* will fall out. Research on the response of *D*. *catenatum* to cold stress is limited; however, the authors found that low temperatures (4 to −2 °C) cause considerable increases in antioxidant activities and electrolyte leakage in *D. catenatum* [22]. GC-MS detection of methanol extract reveals that hydrolysis of sugars, amino acid catabolism, and citrate cycles are all induced under zero temperature treatment [22]. SET DOMAIN GROUP (SDG) proteins mediate chromatin structure modification and transcript regulation, and four SDG members are significantly influenced by low temperatures [23]. Regulation of membrane lipid levels is the major response of biological progress during environmental stresses. Our previous research assessed the response of *D*. *catenatum* to different temperatures. Under freezing conditions, the flow of metabolites pointed to flavonol and anthocyanin biosynthesis pathways [24]. In addition, gene set enrichment analysis revealed that lipid metabolism and photosynthesis are primary processes during FT and FR [24], but lipid species composition and translation regulation are both absent during freezing in previous research. Horticultural and medicinal plants exhibit complex secondary metabolism and regulation mechanisms under environmental stresses. This multi-omics analysis offers new insight into the inter-relationships of the metabolites and transcripts involved in these processes. Previous studies have applied in-depth multi-omics analyses to medicinal plants such as *Isatis indigotica*, *Chimonanthus salicifolius*, *Chimonanthus salicifolius*, *Ginkgo biloba*, *Taxus*, etc., to study their secondary metabolism regulation [25,26,27,28,29]. The function of lipid species during freezing is still unknown in *D*. *catenatum*. In this study, we integrate multi-omics data, including transcriptome, lipidome, and proteome, and elucidate the processes of lipid remodeling in the plant response to freezing.

## 2. Materials and Methods

### 2.1. Plant Materials and Freezing Conditions

Two-year-old cultivated *D*. *catenatum* were grown in soil in the greenhouse of Zhejiang University (Hangzhou, China) under conditions of 25 ± 2 °C (12 h light/12 h dark), 80 μmol photons m^−2^ s^−1^, and 65–75% relative humidity [30]. Freezing treatment conditions were performed according to the previous study [24]. In brief, plants were placed at 0 °C and then treated to a gradual drop from 0 to −6 °C within 3 h. The temperature was held at −6 °C for 3 h. Subsequently, the temperature was gradually raised to 8 °C for 12 h and then held at 8 °C for 12 h as a post-freezing recovery treatment. The soil was treated with ice chips to avoid supercooling of the plants. No illumination was provided during freezing treatment and a light of 30 μmol photons m^−2^ s^−1^ was provided during the recovery phase. All leaf samples were immediately frozen in liquid nitrogen for proteome and lipidome detection.

### 2.2. Proteomic Analysis

The proteomic was analyzed by Metware Biotechnology Co., Ltd. (Wuhan, China). Samples were ground into powder in liquid nitrogen and incubated with an extracted buffer containing 7 M urea, 2 M thiourea, 4% SDS, 40 mM Tris-HCl, pH 8.5, 1 mM PMSF, 2 mM EDTA, and 10 mM DTT for 10 min on ice. The supernatant of lysate was air-dried and resuspended in 500 μLurea (8 M) and tetraethylammonium bromide (100 mM, pH 8.0). The total protein concentration was quantified using a BCA protein assay kit. Proteins (100 μg) of each sample were digested by trypsin at 37 °C for 12 h. Peptides were digested and detected according to a previous study [29]. Briefly, peptides were separated by UHPLC (nanoElute, Bruker Daltonics, Bremen, Germany) with C18-RP columns (5 cm × 75 μm ID, 1.6 μm, IonOpticks, Fitzroy, Australia). The filtrate was separated by a linear gradient of 2% to 80% ACN containing 0.1% formic acid with a flow rate of 0.3 μL min^−1^ and 50 °C column temperature. The HPLC was then coupled online to an MS/MS hybrid, timsTOF Pro2 (Bruker Daltonics, Bremen, Germany), and CaptiveSpray nano-electrospray ion source (Bruker-Michro, Billerica, MA, USA). The capillary voltage was set to 1400 V, and the MS and MS/MS spectra were acquired from 100 to 1700 m/z. As for the ion mobility range (1/K0), 0.7 to 1.4 Vs cm^−2^ was used. The timsTOF accumulation and ramp time were both set to 100 ms, which enabled operation at duty cycles close to 100%. MS raw data were analyzed by FragPipe (v17.1) by searching against the NCBI protein database (taxid_37818).

### 2.3. Lipidomic Analysis

Lipidomics was analyzed by Shanghai Applied Protein Technology Co., Ltd. (Shanghai, China). Lipids were extracted according to a previous study [31]. Briefly, samples were ground into powder in liquid nitrogen and mixed into internal lipid standards with 200 μL water and 240 μL methanol. In total, 800 µL of MTBE were then added into extraction for 30 min at room temperature. After centrifugation, the organic solvent layer was dried under nitrogen. The lipid extracts were re-dissolved in 200 µL 10% ACN/isopropanol, and 3 µL of the sample were injected into UHPLC (Nexera LC-30A, SHIMADZU, Kyoto, Japan) using a CSH C18 column (1.7 µm, 2.1 mm × 100 mm, Waters, Milford, CT, USA). The detection assay was described in a previous study [30]. Briefly, the filtrate was separated by a linear gradient of 30% to 100% ACN/isopropanol (1:9, *v*/*v*) containing 0.1% formic acid and 0.1 mM ammonium formate with a flow rate of 300 μL min^−1^. The ESI parameters of Q-Exactive Plus (Thermo Scientific, Waltham, MA, USA) were set as follows: 300 °C source temperature; 350 °C capillary temperature, 3000 V ion spray voltage, 200–1800 m/z scan range, 50% S-Lens RF level. Lipid species were identified by LipidSearch Software (Thermo Scientific, Waltham, MA, USA) based on the 5 ppm mass tolerance of each fragment and the 5% product ion threshold.

### 2.4. Transcriptome Data Analysis

Gene expression levels of FT and FR were supported by our previous study [24]. The raw data are available from the BIG Data Center of the Chinese Academy of Sciences (http://bigd.big.ac.cn; accessed on 1 September 2022) with accession numbers CRA003229 and CRA005177.

### 2.5. Data Analysis

Proteome and lipidome data were treated by hierarchical clustering using the R package heatmap (v1.0.12) and by PCA using the R package FactoMineR (v2.6). GO enrichment analysis was used in the R package GOplot (v1.0.2) and clusterProfiler (v4.2.2) [31]. For DAPs selection, the protein levels of the two comparisons were determined by FC > 1.5 or FC < 0.7 and with statistical significance (*p*-value < 0.05). DALs of comparisons were selected by FC > 2 or FC < 0.5 with *p*-values < 0.05.

## 3. Results

### 3.1. Effects of Freezing Stress on Lipid Metabolism and Photosynthesis in D. catenatum

**Figure 1:**
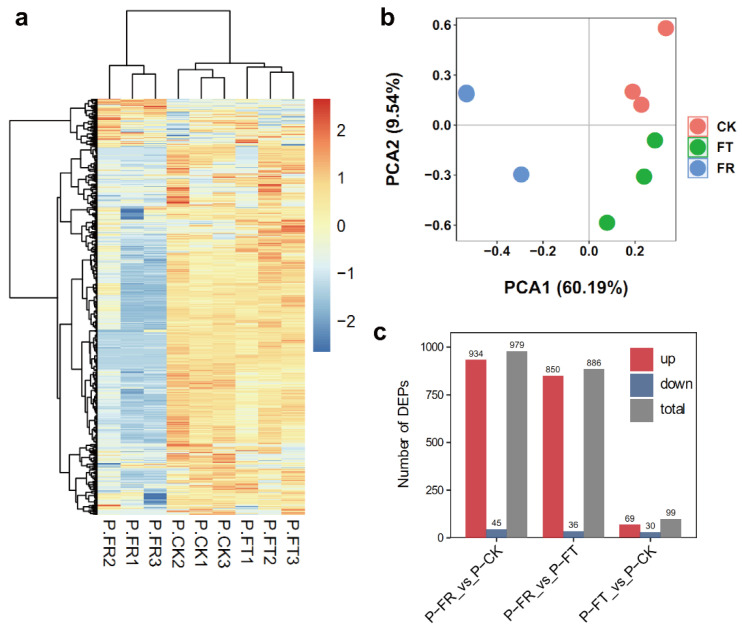
Overview of the proteomes during freezing stress. (**a**) A heatmap of the abundance of proteins in CK, FT, and FR. The bar indicates the significant values. (**b**) PCA analysis of the proteomes in CK, FT, and FR. (**c**) Differentially accumulated proteins (DAPs) in three comparisons.

We previously found that *D*. *catenatum* accumulated more anthocyanin and flavonol under freezing stress conditions [24]. To assess the response of protein levels of *D*. *catenatum* under FT and FR conditions, we carried out proteome analyses with three independent replicates of each treatment. Based on the matched spectra, we identified 6976 proteins, including 5343 quantified proteins (Figure S1). Hierarchical cluster analysis showed that three independent samples formed in a cluster (Figure 1a). Principal component analysis (PCA) showed that the contribution rates of the two treatments were 60.19% and 9.54%, respectively (Figure 1b). Pairwise comparisons of CK (control condition), FT, and FR, identified 99 DAPs in FT vs. CK, 979 DEGs in FR vs. CK, and 886 DEGs in FR vs. FT. We found a significant difference in their expression levels with the threshold of fold change (FC) > 1.5 or <0.7 and *p*-values < 0.05 (Figure 1c).

**Figure 2:**
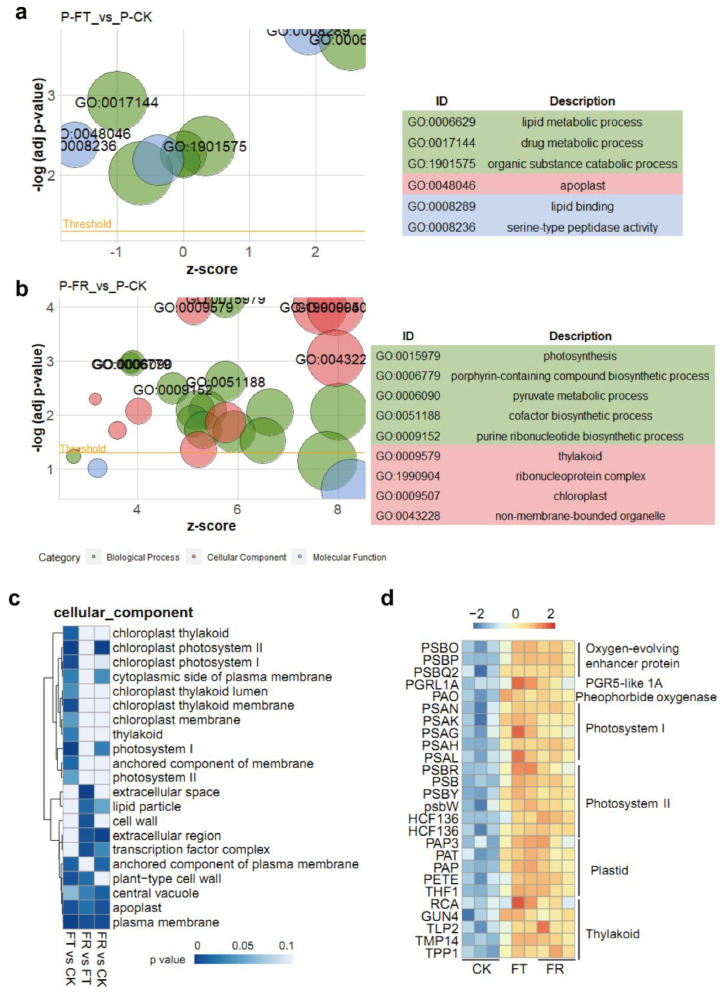
Gene ontology (GO) enrichment analysis of DAPs and DEGs in two comparisons. (**a**) The Go enrichment analysis of DAPs in FT vs. CK. (**b**) The Go enrichment analysis of DAPs in FR vs. CK. The *x*-axis represents the z-score of GO enrichment, while the *y*-axis represents −log_10_ adjusted *p*-value. The dot sizes represent the number of DAPs. GO terms are labeled with a cut-off (adjusted *p*-value < 0.05). (**c**) The cellular component enrichment of DEGs from three comparisons. The bar indicates the *p*-value of KEGG enrichment. (**d**) Heat maps show the abundance of representative transcripts from cellular component enrichment. The enrichment pathway of DEGs is shown on the right. The bar indicates the significant values of gene expression levels. Gene abbreviations are listed in Table S2.

For all identified proteins, we performed gene ontology (GO) annotation, carried out the classification of eukaryotic orthologous groups (KOGs), consulted the Kyoto Encyclopedia of Genes and Genomes (KEGG), predicted subcellular localization, and identified signal peptides (Figure S2). After filtering, we found six GO terms and nine GO terms were significantly enriched (adjusted *p*-value < 0.005) in FT vs. CK, and FR vs. CK, respectively (Figure 2a,b). The majority of the DEGs were significantly enriched in primary metabolism, lipid metabolism, and photosynthesis processes, including the lipid metabolic process GO:0006629, the chloroplast process GO:0009507, and the pyruvate metabolic process GO:0006090. In addition, GO enrichment of differentially expressed genes (DEGs) revealed that ‘cellular component’ terms were associated with chloroplast processes during freezing treatment (Figure 2c). Among these, ‘apoplast’ and ‘plasma membrane’ were significantly enriched in three comparisons (*p*-value < 0.05). Based on hierarchical clustering, the DEGs related to the ‘photosystem’ were significantly upregulated in FT compared with CK (Figure 2d). These results suggested that freezing damage affected both lipid metabolism and photosynthesis of *D*. *catenatum* at the transcriptional and translational levels.

### 3.2. Lipidomic Analysis of FT and FR in D. catenatum

**Figure 3:**
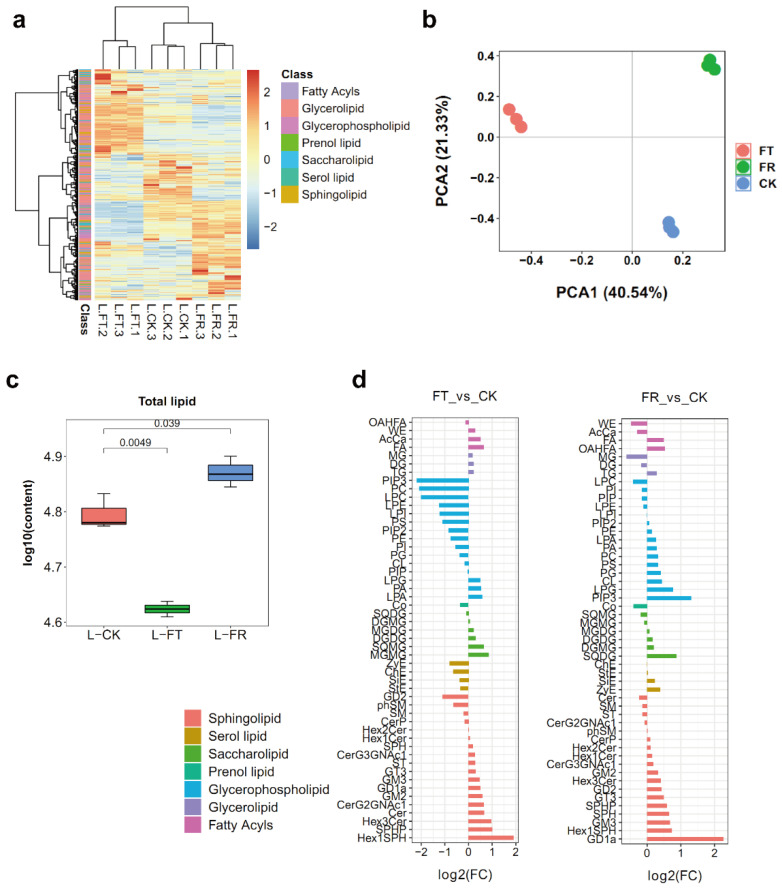
Overview of the lipidomes during freezing stress. (**a**) A heatmap of the abundance of lipid species in CK, FT, and FR. (**b**) PCA analysis of lipidomes in CK, FT, and FR. (**c**) The content of total lipid in CK, FT, and FR. Values are means ± S.D. (*n* = 3); Student’s *t*-test. (**d**) Differentially accumulated lipids (DALs) of two comparisons. Lipid abbreviations are listed in Table S3.

To investigate lipid remodeling under freezing stress, we detected lipid composition in CK, FT, and FR. From the lipidome, we identified seven lipid categories, including 2507 glycerolipids (GLs), 1487 glycerophospholipids (GPs), 996 sphingolipids (SPs), and other lipid classes (Table S1, Figure S3). Based on hierarchical clustering, three separate clusters were found in CK, FT, and FR (Figure 3a). PCA lipidome analysis produced explained values for PC1 and PC2 of 40.54% and 21.33%, respectively (Figure 3b). Total lipid content significantly decreased, by a factor of 1.5, in FT vs. CK, and total lipid content significantly increased, by a factor of 1.2, in FR vs. CK (Figure 3c). We classified 51 differentially accumulated lipids (DALs) into seven categories and displayed their differing accumulation patterns in FT and FR. Most of the sphingolipids were significantly upregulated during both FT and FR (Figure 3d). Phospholipids play critical roles in membrane lipid remodeling under abiotic stresses. The phospholipids were downregulated in FT vs. CK, except for phosphatidic acid (PA), lyso-PA (LPA), and lyso-phosphatidylglycerol (LPG). However, these phospholipids were upregulated in FR vs. CK (Figure 3d), suggesting that membrane lipids degrade during short-term freezing and recover during warming.

### 3.3. Integrated Analysis of Multi-Omics during Freezing Stress

**Figure 4:**
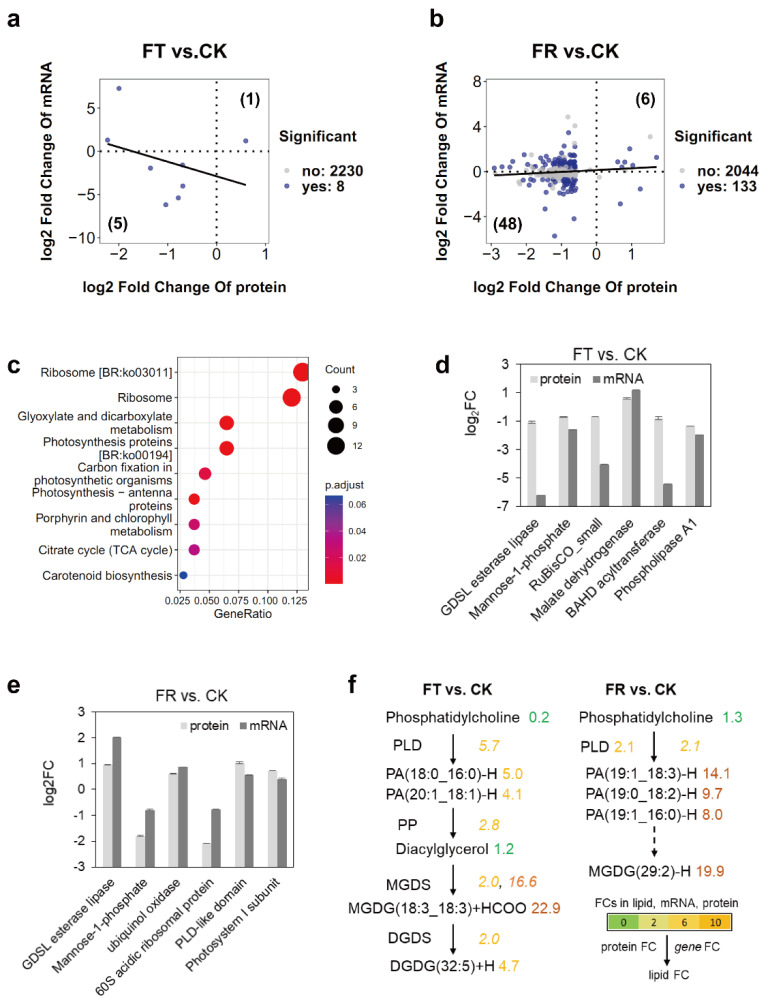
Integrated analysis of multi-omics during freezing stress. (**a**) Scatter plot showing the relationship between changes in protein and mRNA abundances in FT vs. CK. (**b**) Scatter plot showing the relationship between changes in protein and mRNA abundances in FT vs. CK. The colored points indicate significant upregulation or downregulation of protein and mRNA levels. (**c**) KEGG enrichment analysis of mRNAs and encoding proteins based on (**a**,**b**). (**d**) Enrichment of mRNAs and encoding proteins in FT vs. CK. (**e**) Enrichment of mRNAs and encoding proteins in FR vs. CK. (**f**) Simplified lipid biosynthesis flow based on multi-omics. FCs in lipid, mRNA (underlined on the right), and protein levels (left) are listed and highlighted in different colors according to FC values. The ion characteristic fragments and chromatogram of lipids are shown in Figure S5. Abbreviations: DGDS, digalactoslydiacylglycerol synthase; MGDS, monogalactosyldiacylglycerol synthase; PA, phosphatidic acid; PP, phosphatidate phosphatase; PLA, phospholipase A; PLD, phospholipase D.

Previously, we performed transcriptome and untargeted metabolome analyses to study the response of *D*. *catenatum* during freezing [24]. Our transcriptomic analysis suggested that photosynthesis and membrane lipids were affected by freezing stress (Figure 2c). However, the flow of metabolites to these processes remained unclear. In this study, we sought to use multi-omics analysis to determine how metabolic pathways are impacted by freezing at the transcriptional and translation levels. First, we compared values of log_2_ FC for FT vs. CK and FR vs. CK comparisons in the transcriptome and proteome data sets. Of the 2238 combined protein and mRNA values, 8 mRNAs and encoding proteins exhibited differences in expression levels by a factor of at least two in FT vs. CK. Among these genes, six mRNAs and encoding proteins were either upregulated or downregulated (Figure 4a). Moreover, 133 mRNA and encoding protein levels displayed significant variations in FR vs. CK. Among these, 54 mRNAs and encoding proteins were either upregulated or downregulated (Figure 4b). These genes were strikingly enriched in processes related to ribosomes, chloroplasts, and the citrate cycle (adjusted *p*-value < 0.05) (Figure 4c). GDSL esterase lipase and its mRNA levels were downregulated in FT vs. CK and upregulated in FR vs. CK, thus confirming our transcriptomic and proteomic data. Levels of mannose-1-phosphate and its mRNA significantly decreased in both FT vs. CK and FR vs. CK (Figure 4d,e). Two related phospholipid editing proteins, PLA and PLD, were identified in FT vs. CK and FR vs. CK (Figure 4d,e). A significant expression of lipid species (Figure S4) was integrated with the protein and mRNA data (Figure 4f). Total PC content was downregulated five-fold during FT; in particular, PLD, which hydrolyses PC into choline and PA, increased by a factor of 5.7 in FT vs. CK. During FT, we also found significant increases in PA and MGDG levels associated with the upregulation expression of *PP* and *MGDS* by factors of 5.0 for PA (18:0_16:0), 4.1 for PA (20:1_18:1), and 22.9 for MGDG (18:3_18:3). Levels of PA and MGDG also significantly increased during FR, by a factor of 2.1, for both PLD proteins mRNA. These results suggest that lipid metabolism and photosynthesis are primary processes in response to freezing.

## 4. Discussion

Proteomes are used for translational regulation research related to the plant response to cold stress conditions [32,33]. Increased levels of DAPs involved in ROS scavenging, photosynthesis, energy metabolism, and carbohydrate metabolism are thought to enhance tolerance to chilling stress [32], further suggesting that the cold tolerance of plants can be enhanced by cold acclimation. Changes in metabolites and proteins associated with protein homeostasis, carbohydrate metabolism, secondary metabolism, and lipid metabolism occur mostly between 20 °C to 16 °C, with levels of 18% and 50% reported for proteins and metabolites, respectively [33]. However, changes in protein levels during freezing stress are still unclear. Our study provides an enrichment analysis of DAPs for the thawing phase. We found that photosynthesis and chlorophyll biosynthesis processes were significantly enriched in FR vs. CK (Figure 2b). Our transcriptomic analysis also suggested chloroplast and thylakoid damage during FT and FR (Figure 2d). The FR process involves tissue thawing. Proteomic studies in onions have shown that proteins are responsible for re-establishing ion homeostasis and proteostasis, cell-wall remodeling, and reactive oxygen species (ROS) scavenging during the freeze-thaw phase [34]. ROS-induced oxidative damage, which affects photosystem II efficiency and the activities of ROS-scavenging enzymes (SOD, APX, CAT), is associated with this process during the post-thaw period [35]. Because of the antioxidant activity of flavonoids, their accumulation during freezing may serve as a protective mechanism in the chloroplast [36]. The association of flavonoid metabolism with lipid synthase has been previously reported. The rice glycosyltransferase, SQDG synthase, not only synthesizes SQDG from the UDP-sulfoquinovose and DAG in the chloroplast but also participates in flavonoid glycosylation [13,31]. In *Arabidopsis*, SQDG synthase mediates a novel anionic glycolipid synthesis under phosphate starvation [12]. However, the function of SQDG synthase during cold stress is still unknown. 

The integrity of chloroplast membranes is critical for photosynthesis and cellular survival after freezing. Galactolipids are important for maintaining the stability of chloroplast membranes in plants [37]. Levels of MGDG (34:6) and DGDG (36:6) in *Arabidopsis* significantly increase with chilling [38]. The authors [39] found changed levels of galactolipids associated with cold resistance in fruits stored at 0 °C. In this study, we found that MGDG and DGDG levels increased in FT and FR (Figure 3d). Levels of higher-unsaturation MGDG (18:3/18:3) increased by a factor of 22.9 in FT vs. CK, and levels of MGDG (29:2) increased by a factor of 19.9 in FR vs. CK (Figure 4f). We identified five galactolipid synthases in *D*. *catenatum*, and most of these are specifically induced by cold stress conditions [40]. We also found increased levels of four upregulated genes related to PC degradation in FT vs. CK. *PLD* increased by a factor of 5.7, *PP* by a factor of 2.8, *MGDS* by a factor of 16.6, and *DGDS* by a factor of 2.0 (Figure 4f). However, analysis of DEPs revealed no selection of galactolipid synthases in FT vs. CK. The regulation of galactolipids biosynthesis during freezing is a matter worthy of further study. 

Changes in phospholipid content during cold stress periods are important for membrane structure. PLDs are involved in the hydrolysis of phospholipids into PA. In *Arabidopsis*, the authors of ref. [7] found that a deficiency in PLDs impacted increases in PA and declines in PC during freezing and post-freezing periods. In the same genus, the authors of ref. [41] found that enhanced levels of PA reduced the extent of damage caused by ROSs under freezing conditions. In this study, we found decreased levels of most phospholipids during FT. PA, LPA, and LPG were notable exceptions. Levels of these phospholipids increased in FR vs. CK and played more positive roles in the recovery phase (Figure 3d). Another important phospholipid, PC, is regarded as a biomarker of cold stress. The remodeling of phospholipids during stress involves rapid degradation of PC [7,42,43]. Downregulation of PLA and upregulation of PLD protein levels might be directly correlated with the changes in PC levels which we found in FT and FR, respectively (Figure 4f). By such means, PC metabolic changes might alleviate the damage caused during freezing. 

## 5. Conclusions

Studies of transcriptome and metabolome reprogramming associated with freezing have revealed a gene-to-metabolite network in *D*. *catenatum* [24]. The remodeling of phospholipid and galactolipid plays an important role during the freezing and recovery phase, including C34:0-PA, C38:2-PA, C36:6-MDGD, and C32:5-DGDG. Our proteome analysis further confirms the flow of biological information at the protein level during the freezing and recovery phase. The candidate genes/proteins of the marked pathways indicate better means of enhancing cold tolerance in *D*. *catenatum*. However, we still lack information on gene regulation during freezing relating to such processes as post-translational modification. As more multi-omics data emerge and better-integrated tools are produced, future multi-omics studies will help in bridging the gap from genotype to phenotype in medicinal plant research.

## Data Availability

The data presented in this study are available in BIG Data Center, accession numbers (CRA003229 and CRA005177).

## Supplementary Materials

The following supporting information can be downloaded at: https://www.mdpi.com/article/10.3390/metabo12121216/s1. Figure S1: Quality control of the proteome. Figure S2: The annotations of total proteins. Figure S3: Seven subgroups of lipids are detected using lipidomics. Figure S4: The lipid species of DELs in two comparisons. Figure S5: The ion characteristic fragments and chromatogram for eight lipid species. Table S1: Lipidomics classification during freezing. Table S2: Gene abbreviations list. Table S3: Lipid abbreviations list.
