# Supplementary material for: Dendrobium Multi-Omics Reveal Lipid Remodeling in Response to Freezing
Source: Metabolites. 2022 Dec 3;12(12):1216. doi: 10.3390/metabo12121216 (PMC9784835; doi:10.3390/metabo12121216)
Supplement: Supplementary file 1 [file metabolites-12-01216-s001.zip › metabolites-2074083-supplementary/suppl_Figure.pptx]

## Slide 1
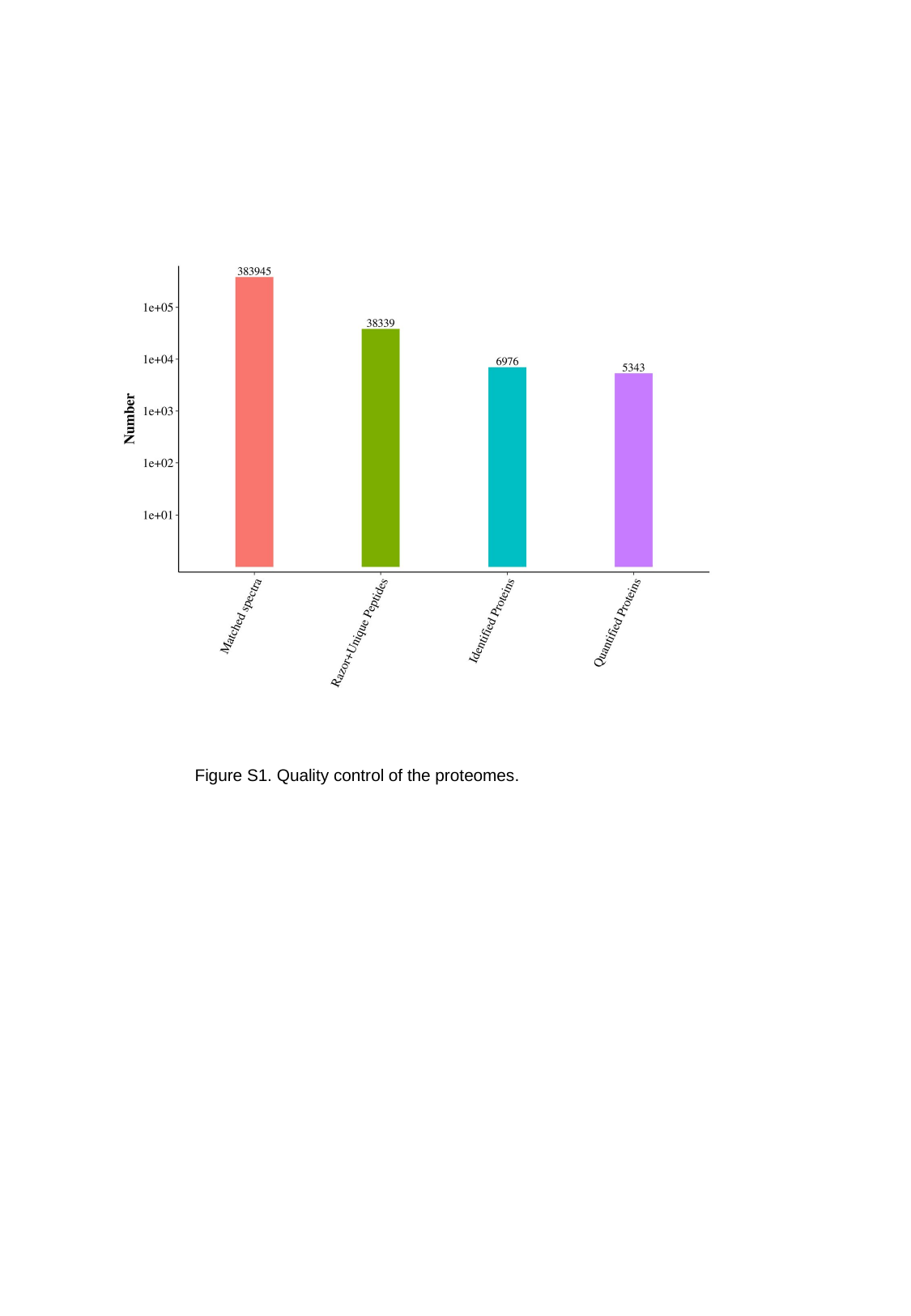

Figure S1. Quality control of the proteomes.

## Slide 2
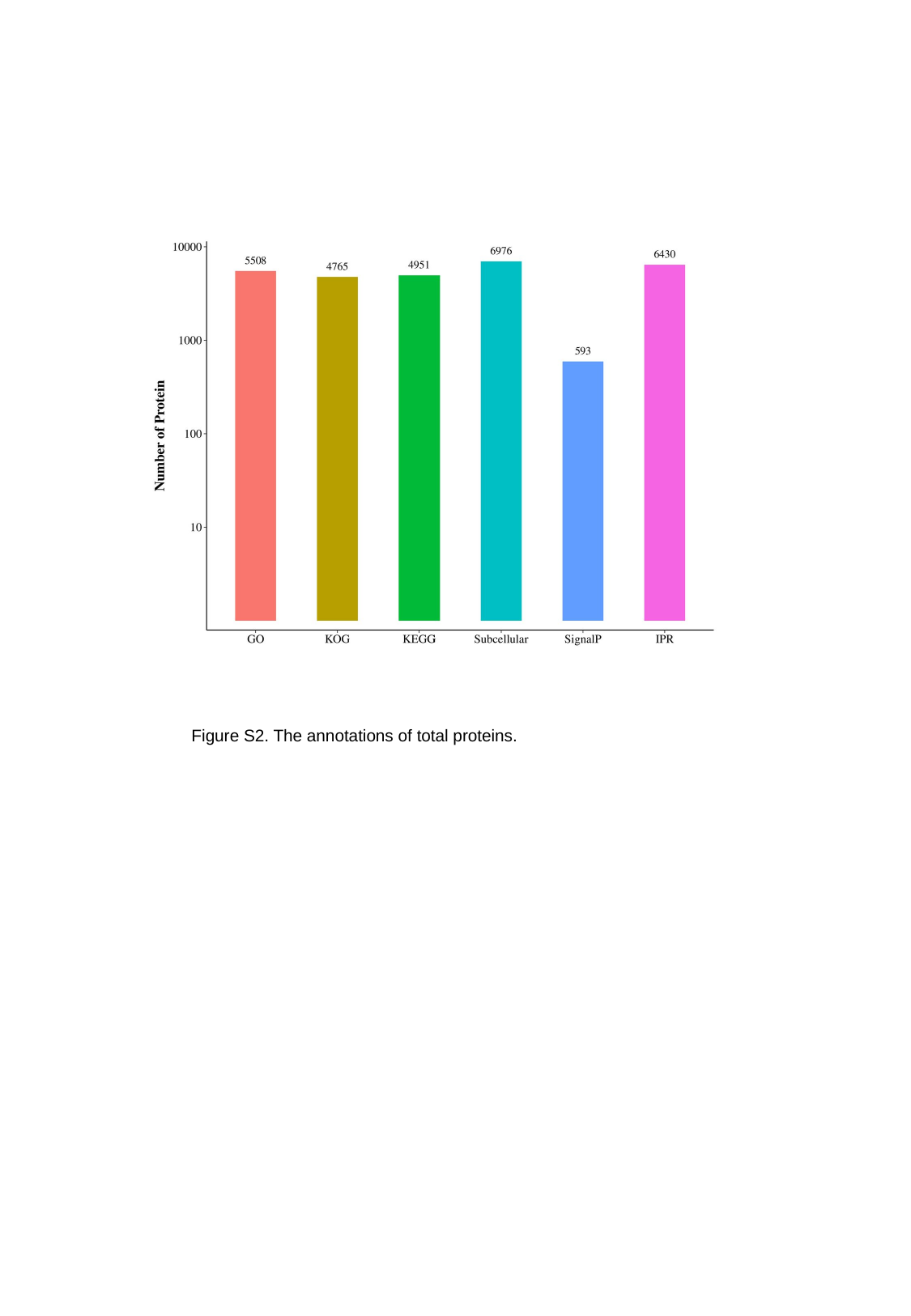

Figure S2. The annotations of total proteins.

## Slide 3
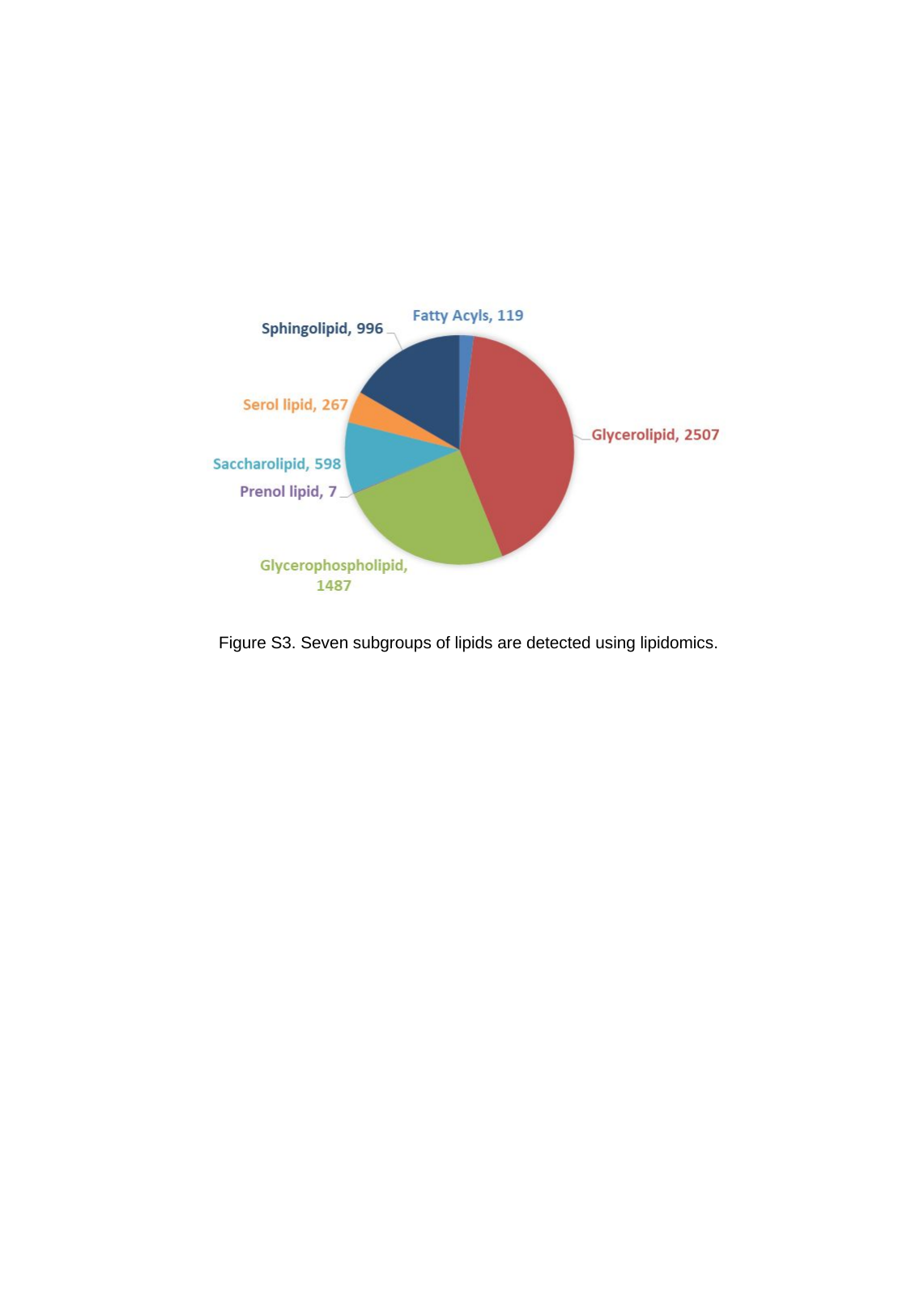

Figure S3. Seven subgroups of lipids are detected using lipidomics.

## Slide 4
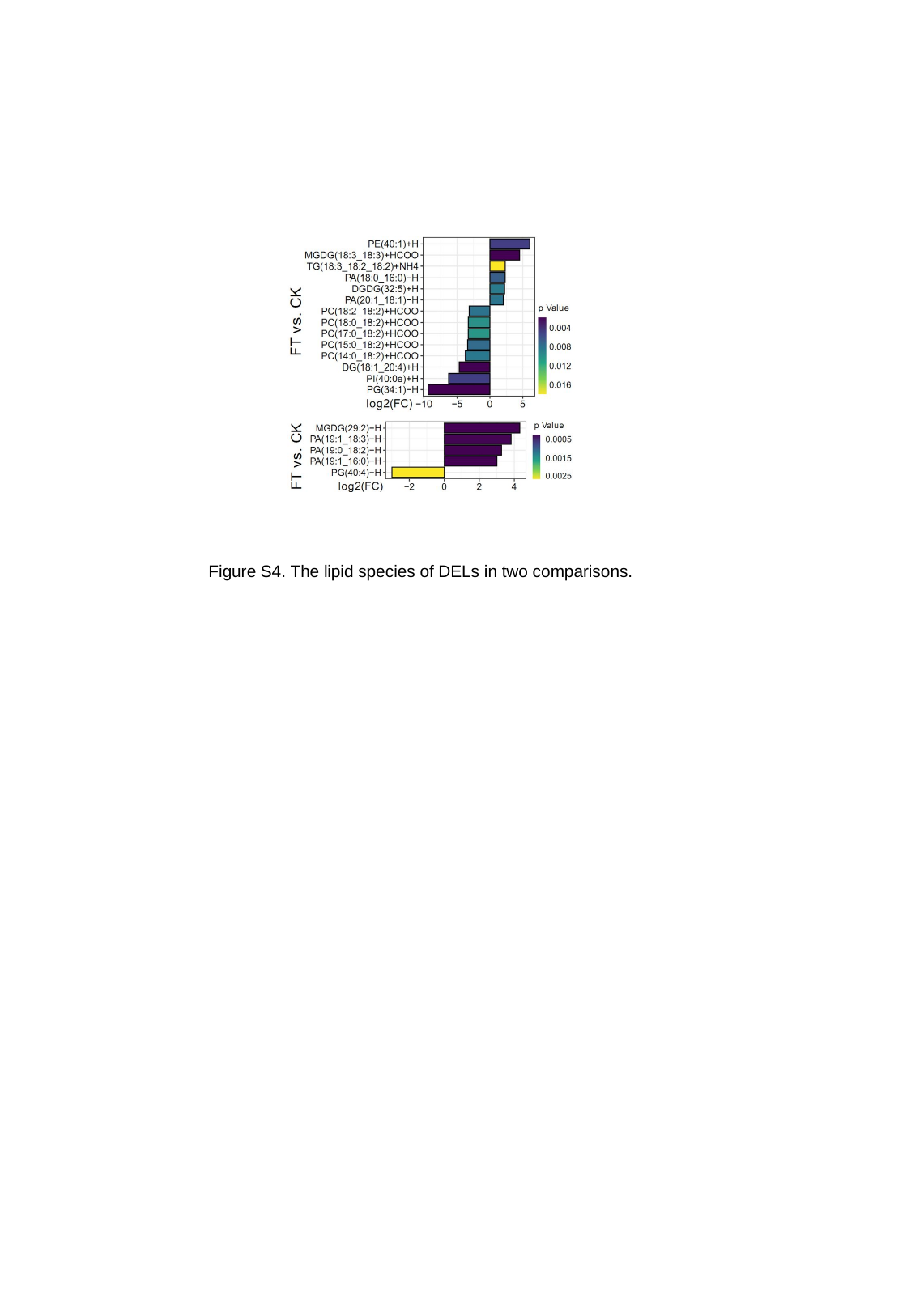

Figure S4. The lipid species of DELs in two comparisons.

## Slide 5
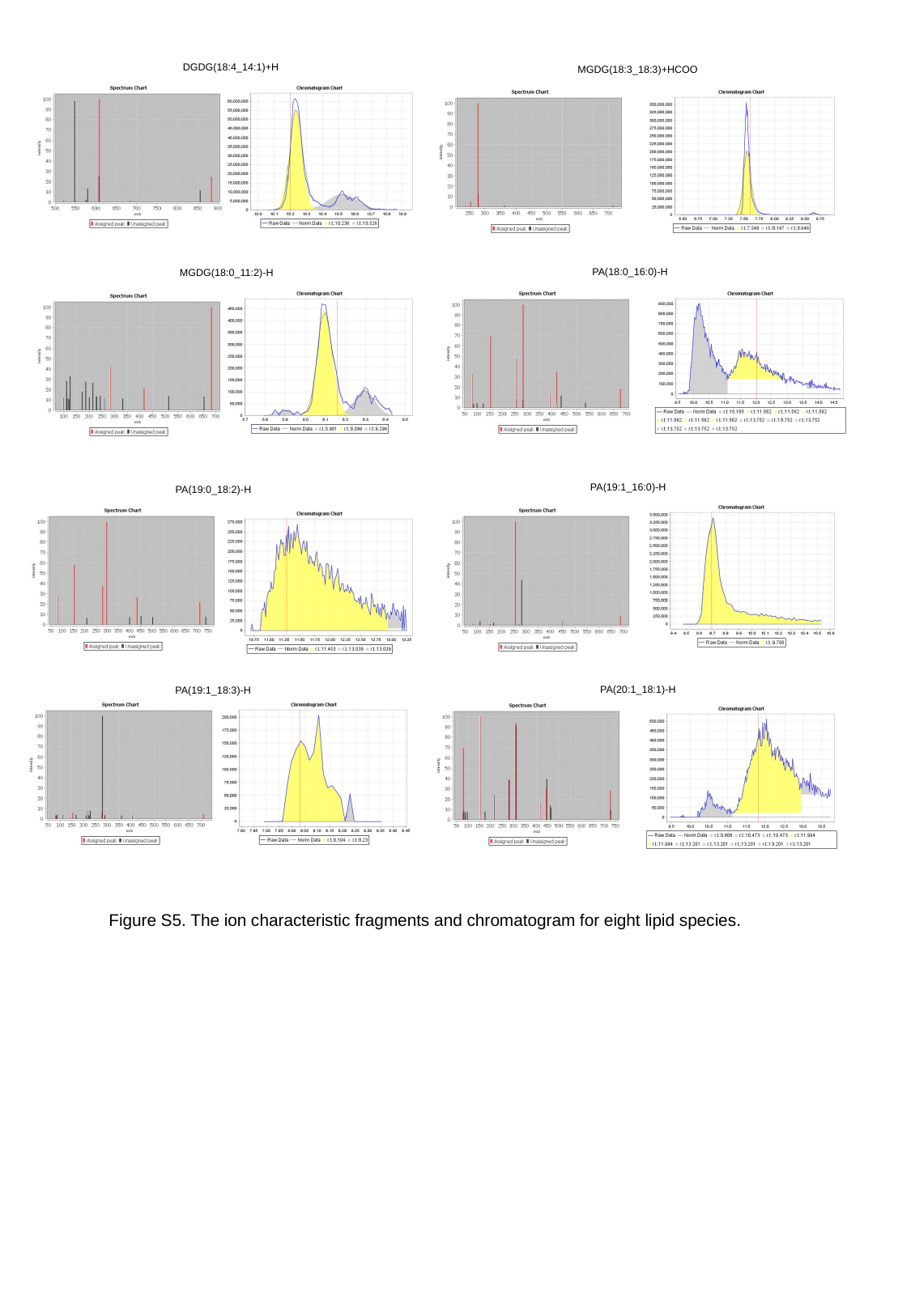

DGDG(18:4_14:1)+H
MGDG(18:3_18:3)+HCOO
PA(18:0_16:0)-H
MGDG(18:0_11:2)-H
PA(19:1_16:0)-H
PA(19:0_18:2)-H
PA(20:1_18:1)-H
PA(19:1_18:3)-H
Figure S5. The ion characteristic fragments and chromatogram for eight lipid species.
